# Lizards and amphisbaenians (Reptilia, Squamata) from the middle Eocene of Mazaterón (Soria, Spain)

**DOI:** 10.1002/ar.25271

**Published:** 2023-06-07

**Authors:** Arnau Bolet

**Affiliations:** ^1^ Departamento de Estratigrafía y Paleontología Universidad de Granada Granada Spain; ^2^ Institut Català de Paleontologia Miquel Crusafont, Universitat Autònoma de Barcelona Barcelona Spain; ^3^ School of Earth Sciences University of Bristol Bristol UK

**Keywords:** Amphisbaenia, Eocene, fossil lizards, Reptilia, Squamata

## Abstract

The assemblage of lizards and amphisbaenians (Reptilia, Squamata) from the middle Eocene locality of Mazaterón (Spain) is described. Considering the rather limited material available for the study, the assemblage shows a moderate diversity with eight taxa corresponding to five different families. In most cases the scarcity and fragmentary nature of squamate specimens precludes a precise identification, but provides insights on identity of the groups represented. Mazaterón fills the gap between early and late Eocene Iberian localities, showing the persistence of iguanids (possibly *Geiseltaliellus*), lacertids (possibly *Dormaalisaurus*), and glyptosaur (tribes glyptosaurini and “melanosaurini”) and anguine anguids through most of the Iberian Eocene. It also records the return of amphisbaenians (Blanidae) after their temporary retrieval from Europe during most of the middle Eocene, and the presence of two scincids, one of them possibly corresponding to a new taxon. The information provided by squamates complements what is already known from mammals, crocodylians, and turtles in what is arguably one of the most important vertebrate Paleogene localities of the Iberian Peninsula.

## 1 INTRODUCTION

The Iberian middle Eocene locality of Mazaterón has yielded an abundant and highly diverse vertebrate paleofauna containing macro and micro vertebrates, with Guisado et al. (1988), Cuesta Ruiz‐Colmenares (1988, 1991), Jiménez Fuentes et al. (1989), and Gil Tudanca (1992) as the starting point of research. Described vertebrates so far include mammals like perissodactyls (e.g., Badiola et al., 2009, 2022; Badiola & Cuesta, 2008; Cuesta Ruiz‐Colmenares, 1988, 1993a, 1996; Jiménez Fuentes et al., 1989; Perales‐Gogenola et al., 2022), artiodactyls (e.g., Cuesta & Badiola, 2009; Cuesta Ruiz‐Colmenares, 1993b), creodontans (Cuesta Ruiz‐Colmenares, 1992), rodents (Peláez‐Campomanes, 1996), and primates (Marigó et al., 2010; Minwer‐Barakat et al., 2012; Moyà‐Solà & Kohler, 1992), as well as remains of large members of the paleoherpetofauna like turtles and crocodiles (Cuesta Ruiz‐Colmenares & Jiménez‐Fuentes, 1994; Jiménez Fuentes et al., 1989; Ortega et al., 2022; Pérez‐García et al., 2016). The forms of the latter currently recognized at Mazaterón include three different turtles (Podocnemididae, Trionychidae, and Testudinidae) and two crocodyliforms corresponding to a notosuchian close to *Iberosuchus* and the alligatoroid crocodilian *Diplocynodon* (Ortega et al., 2022). Despite the existence of early studies on other microvertebrates like rodents and primates, small members of the paleoherpetofauna have remained unstudied.

Here the assemblage of lizards and amphisbaenians from Mazaterón is described for the first time, based on the collection stored at the Institut Català de Paleontologia Miquel Crusafont (see Section 3). Amphibians and snakes are scarce but present in the collection and will be described elsewhere (work in progress). Besides providing a more complete view of the vertebrate assemblage, the study of the lizards and amphisbaenians from Mazaterón is important in that these represent, together with a preliminary account of the assemblage from the roughly contemporaneous Catalan locality of Pontils (Minwer‐Barakat et al., 2023), the first middle Eocene squamates ever described from the Iberian Peninsula. Together, these two localities fill a gap between the known faunas from the Iberian early Eocene (Portugal, Rage & Augé, 2003; Catalonia, Bolet, 2017) and late Eocene (Catalonia, Bolet & Evans, 2013; Bolet & Augé, 2014). It is also worth noting that these Eocene Iberian localities comprise almost all that is known about Paleogene lizard assemblages in the southern peninsulae of Europe, because equivalent faunas are unknown in the Italian Peninsula and Greece (but see Georgalis, Čerňanský, & Mayda, 2021 for Oligocene localities from southern Balkans). Cuesta Ruiz‐Colmenares and Jiménez‐Fuentes (1994) provided a comprehensive account of the vertebrates of Mazaterón. However, although numerous studies have updated our knowledge of the fauna present in the site (see list in Minwer‐Barakat et al., 2012, to which subsequent additions can be found in Ortega et al., 2022, Perales‐Gogenola et al., 2022 and Badiola et al., 2022), the brief mention cf. Lacertidae in Cuesta Ruiz‐Colmenares and Jiménez‐Fuentes (1994), without any description or figure, is all that was previously known for squamates from the locality. The material reported herein was briefly mentioned in Bolet and Evans (2010a), but a description and identification of the material, and discussion of the results, is provided here for the first time.

## 2 GEOLOGICAL SETTING

The site of Mazaterón is nearby the town of the same name, situated in the Almazán Basin, the geology and stratigraphy of which has been described, among others, in Armenteros (1994) and Huerta and Armenteros (2006). Of the four depositional sequences, Mazaterón is situated in the lowest one, representing the oldest fossiliferous level of the basin, the level containing vertebrate remains belonging to the lacustrine sediments deposited in the central anoxic bottom of a lake system with permanently inundated central areas (Huerta & Armenteros, 2006) corresponding to the Mazaterón Formation. The mammal fossil assemblage, one of the richest of the Duero Basin, has been traditionally assigned to the MP15–16 Paleogene reference level (e.g., Cuesta Ruiz‐Colmenares, 1991; Cuesta Ruiz‐Colmenares & Jiménez‐Fuentes, 1994; Pelaez‐Campomanes, 1996), but it has been recently regarded as corresponding to the MP16 (Badiola et al., 2022).

## 3 MATERIALS AND METHODS

All the fossil remains included in this study are housed at the collections of the Institut Català de Paleontologia in Sabadell (Catalonia, Spain). Specimens were recovered in 1991 from the Mazaterón locality (Almazán Basin, Soria, Spain), using screen‐washing methods. As is usually the case of material that source from screen‐washing, the process usually fragments the specimens that would have been otherwise completely preserved as isolated elements. Some specimens show signs of digestion or other processes that altered their original appearance. Morphological terminology follows Richter (1994), Kosma (2004), and Evans (2008).

## 4 RESULTS

### 4.1 Systematic paleontology

Squamata Oppel, 1811

Scinciformata Vidal and Hedges, 2005

Scincidae Gray, 1825 sensu Estes et al., 1988

cf. Scincidae indet. Morphotype 1.

Material: IPS128814, partial right maxilla.

**FIGURE 1:**
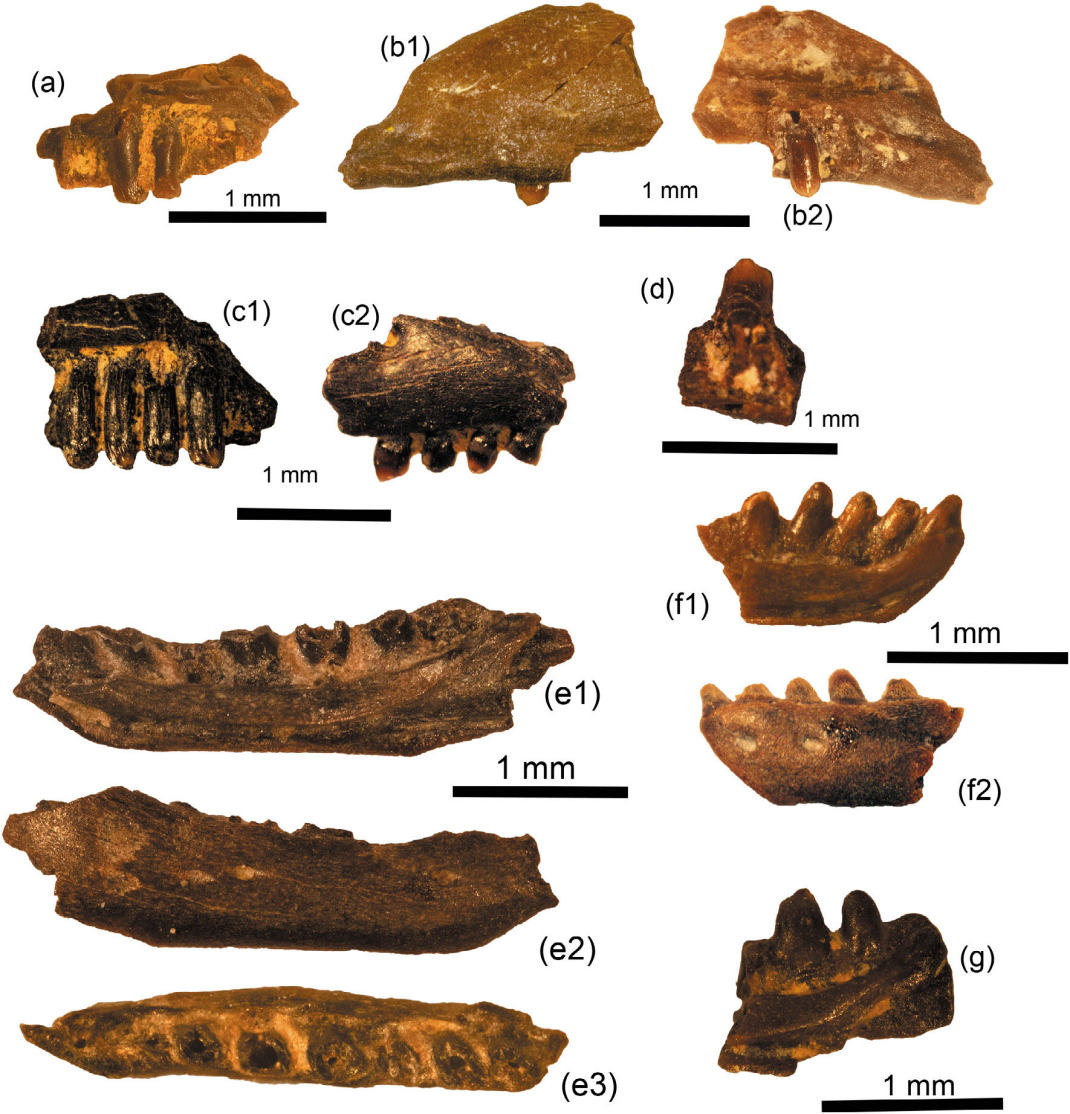
Scinciformatans, lacertids, and amphisbaenians from the middle Eocene locality of Mazaterón (Spain). (a) cf. Scincidae indet. Morph. 1: fragment of right maxilla (IPS128814) in lingual view; (b) cf. Scincidae indet. Morph. 2: fragment of right maxilla (IPS128800) in lingual (b1) and labial (b2) views; (c) Lacertidae indet.: fragment of right maxilla (IPS128801) in lingual (c1) and labial (c2) views; (d) cf. Lacertidae indet.: tooth attached to bone (IPS128813) in lingual view; (e) cf. Blanidae indet.: right dentary (IPS49832) in lingual (e1), labial (e2), and occlusal views (e3). (f) cf. Blanidae indet.: anterior portion of left dentary (IPS128804) in lingual (f1) and labial (f2) views. (g) cf. Blanidae indet.: posterior portion of right dentary (IPS128799) in labial view.

Description: Scinciformata and, more tentatively, scincids, are represented at Mazaterón by two different morphotypes presenting highly differentiated tooth crown morphologies. IPS128814 (Figure 1a) is a right maxilla that is described as morphotype 1. The dentition is characterized by pleurodont, robust, and columnar teeth with an apparently variably constrained neck. The teeth are lingually slightly concave, and the tooth shaft is apparently slightly curved, at least in its posterior margin. The best‐preserved tooth has a transversely bicuspid crown, presenting a cuspis labialis and a smaller cuspis lingualis, and a faint lingual striation. This crown also shows that the cuspis labialis and lingualis are separated by a well‐developed antrum intercristatum that is very narrow between the cusps, but widens both anteriorly and posteriorly. The tooth shaft is strongly asymmetric, with a crista mesialis that is longer than the crista distalis, forming a mesially expanded crown. The crista lingualis anterior and posterior are, on the other hand, equally long. The supradental shelf is well‐developed, being both robust and extended in medial direction. The labial surface of the bone is smooth, lacking any trace of ornamentation or osteodermal crust, at least in the preserved portion.

### 4.2 Remarks

The presence of transversely bicuspid teeth (cuspis labialis and cuspis lingualis) and a well‐developed antrum intercristatum is widespread among scincids, but dentition of this morphotype differs from the generalized scincid morphology (see morphotype 2), where the tooth is more symmetrical as the result of the crista mesialis and distalis being almost equally long. The crown morphology of IPS128814 is similar to that of the some herbivorous scincids (e.g., *Egernia cunninghami*), but it is also reminiscent of for example, the cordylid *Cordylus cordylus* (see Kosma, 2004). Referral to scincids is favored in front of cordylids mainly on the basis of the well‐developed antrum intercristatum, but regarded as tentative.

cf. Scincidae indet. Morphotype 2.

Material: IPS128800, fragment of right maxilla.

Description: This morphotype is represented by a posterior fragment of right maxilla preserving one tooth (IPS128800, Figure 1b). This specimen preserves the posteroventral process and part of the nasal process. In lingual view, and situated anterodorsally to the former, there is a long facet most probably for the articulation of the jugal and/or ectopterygoid. On the labial side, the surface of the bone is completely smooth, without any trace of ornamentation or osteodermal crust, and lacking foramina. The supradental shelf is poorly preserved, but it seems to be arched, its posterior portion bending ventrally. The preserved tooth is columnar with straight lateral margins. It surpasses the dental wall by less than one third of its height, and it bears a nutrient foramen situated lingually. The tooth crown shows the typical scincid morphology, being rather symmetrical, with equally long crista mesialis and distalis and equally short crista lingualis anterior and posterior, and a well‐developed antrum intercristatum. It seems to lack well developed striae.

### 4.3 Remarks

The observed tooth morphology, mainly regarding the presence of a well‐developed antrum intercristatum, points toward a generalized scincid, but does not allow a precise identification. Because the preserved tooth belongs to the end of the tooth row it is unlikely that the generalized dentition of this specimen corresponded to a different position in the dental row of the same taxon for which morphotype 1 has been described above. The presence of two members of Scinciformata and, among them, possibly two scincids is regarded as the most plausible situation in Mazaterón, although additional specimens are needed to justify a more definitive identification.

Laterata Vidal and Hedges, 2005

Lacertibaenia Vidal and Hedges, 2005

Lacertidae Oppel, 1811

Lacertidae indet.

Material: IPS128801, fragment of right maxilla; IPS128813, tooth attached to bone.

Description: Only the recovery of fragments of dentition with mesiodistally bicuspid teeth hints at the presence of lacertids. More specifically, IPS128801 (Figure 1c) is a fragment of right maxilla presenting apparently unicuspid teeth anteriorly, but developing a rather rounded accessory cusp situated mesially to the main cusp in the posteriormost preserved tooth. The crown presents, in its lingual side, few but well‐developed striations. The preserved teeth belong to the anterior portion of the tooth row (as evidenced by the preserved ventral margin of the external naris on the dorsal margin of the bone), so it seems that only anteriormost teeth would have been unicuspid. It is not possible to state if posteriormost teeth were bicuspid or tricuspid, because this portion of the tooth row is not preserved. Teeth are columnar and straight, only the crowns (not the necks) are bent posteriorly. Labially, the external surface of the bone is smooth (at least in the preserved portion) and bears a large labial foramen. IPS128813 (Figure 1d) is a tooth attached to an indeterminate tooth‐bearing bone (maxilla or dentary). It also shows some bicuspidity, but morphology is apparently different from that of IPS128801 in that the main cusp is smaller, and the accessory cusp is less distinct.

### 4.4 Remarks

The morphology of the bicuspid tooth in IPS IPS128801 is compatible with that of a lacertid with poorly developed bicuspidity (rounded small mesial cusp). More specifically, among known Eocene lacertids, its size, developed striation, and poorly developed second cusp in bicuspid teeth compares well with *Dormaalisaurus*, which was also identified from the late Eocene Iberian locality of Sossís (Bolet & Evans, 2013). A possible lacertid, that was regarded as potentially similar to *Dormaalisaurus*, was also identified at the early Eocene of Catalonia (Bolet, 2017). This genus is also known from the early Eocene of Belgium and France and middle Eocene of France (Augé, 2003a; Augé & Smith, 2002), but the fragmentary nature of the material at hand precludes a referral to the genus. The identification of a different bicuspid tooth morphology could be hinting at the presence of a second lacertid, although again material is too scarce and poorly preserved to be certain about this. The tooth presents a morphology that is clearly different from that of the specimen referred to *Dormaalisaurus*. It could belong to a second lacertid or, less likely, to an eolacertid, the fossil sister group of lacertids (Čerňanský & Smith, 2018, 2019) that has Eocene representatives like *Eolacerta*, and which sometimes present a poorly developed accessory cusp.

cf. Lacertidae indet.

A morphotype of vertebra (IPS128808) is clearly different from that described below and assigned to indeterminate anguines. This morphotype is characterized by a small centrum that is much wider than the condyle width at the point where both elements contact.

### 4.5 Remarks

**FIGURE 2:**
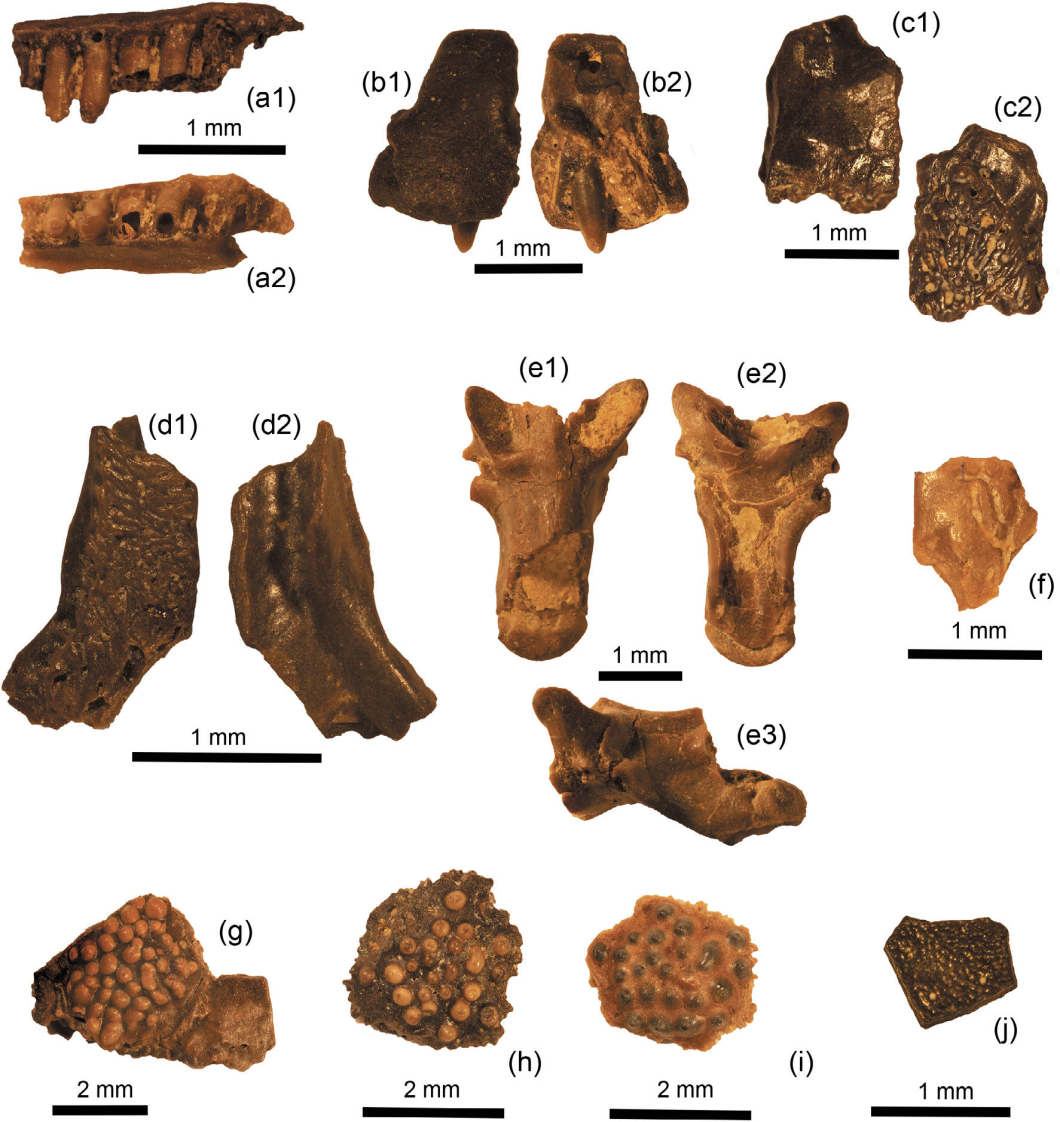
Iguanians and anguimorphs from the middle Eocene locality of Mazaterón (Spain). (a) Iguanidae indet: fragment of maxilla (IPS128802) in lingual (a1) and labial (a2) views; (b) Anguidae indet.: partial premaxilla (IPS128803); (c) Anguinae indet., partial right ?nasal (IPS128798) in ventral (c1) and dorsal (c2) views; (d) Anguinae indet., partial left frontal (IPS49838) in dorsal (d1) and ventral (d2) views; (e) Anguinae indet.: partial caudal vertebra (IPS128807) in dorsal (e1), ventral (e2), and lateral (e3) views; (f) Anguinae indet., partial body osteoderm (IPS128809); (g–i) Glyptosaurini indet.: cranial osteoderms (IPS49833‐49835); (j) cf. “Melanosaurini” indet., cranial osteoderm (IPS128809).

The wide centrum (in comparison to the base of the condyle) is characteristic of clades formerly grouped in the now considered paraphyletic “Scincomorpha,” and that classically contained “scincoids” (scincids and cordyliforms) and lacertoids. It strongly contrasts with vertebrae presenting centra that show a continuous lateral shaft between the centrum and condyle. The latter feature is characteristic of anguid vertebrae, as exemplified by the vertebrae described below (Figure 2e). It is tentatively referred to an indeterminate lacertid, although it cannot be ruled out that it belonged to a scincoid.

Amphisbaenia Gray, 1844

Blanidae Kearney, 2003

cf. Blanidae indet.

Material: IPS49832, right dentary (Figure 1e); IPS128804, anterior portion of left dentary (Figure 1f) and IPS128799, posterior portion of right dentary (Figure 1g).

Description: Amphisbaenians are represented at Mazaterón by three fragmentary dentaries (Figure 1e–g). One of them (IPS49832, Figure 1e) shows the general shape of this element, lacking only the tooth crowns, which were most probably broken during the screen‐washing process, and its anterior and posteriormost tips. The dentary is short and robust, bearing a Meckelian fossa that is open for its entire length and oriented lingually. The ventral margin of the dentary is straight, except for an angle formed at the level between the two anteriormost teeth and the symphyseal portion. The subdental shelf is well‐developed, with a slightly concave arched morphology. The shelf becomes thinner toward the posterior end, resulting in a crescent appearance. A weak sulcus dentalis is present. A fused intramandibular septum is preserved. Teeth have a pleurodont implantation, with a count of seven, and have an elliptical section with an anterolabial to posterolingual orientation. This specimen provides limited information on tooth morphology, other than its heterodonty in terms of base size, with the first tooth being the one with the largest base. IPS128804 (Figure 1f) and IPS128799 (Figure 1g) are additional dentary portions showing the typical morphology of blanids, with conical, unicuspid and simple tooth crowns. It is worth noting that the rather rounded (instead of pointed) morphology of these tooth crowns is likely related to a process of digestion. Even after this process that has clearly changed the tooth appearance, at least one of the crowns shows a lingual concavity (fourth tooth in IPS128804). IPS128799 shows a circular basal foramen at the base of the last tooth. In IPS49832, there are two preserved labial foramina, but a third one was likely situated on the anterior broken margin of the bone. This is confirmed through observation of the labial side of the anterior portion of dentary IPS128804, that contains two closely situated foramina.

### 4.6 Remarks

Among known amphisbaenians, it can be excluded that the small amphisbaenian from Mazaterón belonged to Rhineuridae or Trogonophiidae on the basis of the open Meckelian fossa and the pleurodont dentition, respectively (e.g., Augé, 2012; Gans, 1960). Of the fossil European amphisbaenians described so far, the material from Mazaterón differs from polyodontobaenids (Folie et al., 2013) in presenting a lower tooth count, an angle at the mandibular symphysis, a rather straight ventral margin of the dentary, and a dentition that does not present a gradual decreasing size toward the anterior end of the bone. The Eocene genera *Louisamphisbaena* Augé, 2012 and *Cuvieribaena* Schleich et al., 2015 and the Oligocene‐Miocene *Palaeoblanus* Schleich, 1988, seem to share with IPS49832 from Mazaterón the presence of an enlarged first tooth in the dentary. Note, however, that the other anterior portion from Mazaterón (IPS128804) preserving this region does not share this character, pointing to a possible variability, and that other characters seem to preclude a referral to any of these genera. For example, *Louisamphisbaena*, from the middle Eocene of France (Augé, 2012), shows a dentary that is more slender and a dentition that is more widely spaced; *Cuvieribaena*, from the middle Eocene of France (Čerňanský et al., 2015) bears a lower number of dentary teeth (six); and *Palaeoblanus*, from the late Oligocene to early Miocene of Germany (Schleich, 1988) shows a more homodont dentition. *Blanosaurus* Folie et al., 2013, from the early Eocene of Belgium and France, differs from the material from Mazaterón in presenting more robust teeth with wider bases and a more homodont dentition, and it also lacks the enlarged first tooth. *Anniealexandria* Smith, 2009, first described from the USA, has been also reported from Europe (Augé, 2012), although this referral, that was mainly based on the presence of nine dentary teeth, has been questioned (Bolet et al., 2014) because other genera, like, for example, *Blanus*, can occasionally present up to nine teeth. The generalized dentary and tooth morphology of the material from Mazaterón is reminiscent of blanids and amphisbaenids, of which only blanids have been recovered in Europe. Moreover, the morphology of this material compares well with the Iberian late Eocene amphisbaenian dentary from Sossís, which was referred to cf. Blanidae indet. (Bolet & Evans, 2013).

Toxicofera Vidal & Hedges, 2005

Iguania Cope, 1864

Iguanidae Bell, 1825

Iguanidae indet.

Material: IPS128802, fragment of maxilla (Figure 2a); IPS49839, tooth‐bearing bone.

The best‐preserved specimen referred to an iguanid is a small fragment of maxilla (IPS128802, Figure 2a) preserving two teeth and the bases of four additional teeth. Tooth implantation is clearly pleurodont, and teeth are columnar, with a neck that is wider than the base and the crown of the tooth. One tooth clearly shows that the condition of the crowns is tricuspid, with a main central cusp and two symmetrically positioned (anterior and posterior) cusplets, separated from the main cusps by longitudinal grooves. At least two teeth show small pits at their bases. A well‐developed supradental shelf is lacking. On the upper surface of the bone, there is a small foramen (superior dental foramen) that is followed by a long and narrow longitudinal groove. IPS49839 is a tooth‐bearing bone with two tall tricuspid teeth that compare well with that described above, although they are clearly higher.

### 4.7 Remarks

Although the morphology of the teeth compares well to the posterior teeth of the widespread iguanid *Geiseltaliellus* (Augé, 2005; Kuhn, 1944) the fact that the anterior dentition is not available does not allow discarding the possibility that this maxilla belonged to the contemporaneous genus *Pseudolacerta*, which mainly differ in showing a caniniform enlarged anterior dentition in both the dentary and maxilla (Augé, 2005; De Stefano, 1903). Dentaries of *Cadurciguana*, the third European Eocene iguanid, are usually identified by the presence of a closed and fused Meckelian fossa (Augé, 1987, 2005). It is much more difficult, however, to differentiate maxillae of *Cadurciguana* from those of *Geiseltaliellus*, because of the lack of diagnostic characters in this element. Only *Geiseltaliellus* has been recognized in the Iberian early Eocene (Bolet, 2017; Rage & Augé, 2003), but both *Geiseltaliellus* and *Pseudolacerta* are known from the late Eocene of Sossís (Bolet & Evans, 2013). *Geiseltaliellus* has been tentatively identified at the middle Eocene locality of Pontils (Minwer‐Barakat et al., 2023), and the dentition of a second specimen (IPS49839) particularly resembles *Geiseltaliellus pradiguensis* in its exceptionally tall pleurodont tricuspid teeth projecting only slightly above the parapet of the dentary. The presence of *Geiseltaliellus* in the Iberian middle Eocene is thus regarded as probable and, allowing for the gaps in available localities, hints at a continuous presence of this iguanian through the Eocene of this region, until the MP17a of Sossís at the very least.

Anguimorpha Fürbringer, 1900

Anguidae Gray, 1825

Anguinae Gray, 1825

Anguinae indet.

Material: IPS128798, partial skull bone (Figure 2c); IPS49838, partial left frontal bone (Figure 2d); IPS128807, a partial caudal vertebra (Figure 2e); IPS128809 (Figure 2f), fragment of osteoderm; IPS49836, and IPS49840 (fragmentary body osteoderms); IPS49844, a fragmentary vertebra; IPS49841, a tooth‐bearing bone with one broken tooth.

Description: Anguine lizards are clearly represented by small partial skull bones, fragmentary body osteoderms, vertebrae, and a tooth‐bearing bone. The two portions of skull bone are incomplete, but IPS49838 is identified as a partial left frontal, and IPS128798 could represent the right counterpart of this same element or a right nasal. Both elements share the same ornamentation formed by a vermiculate osteodermal crust (formed by grooves and pits) fused to the dorsal part of the bone. The left frontal preserves its middle and posterolateral portions. The element shows an osteodermal crust (shield) running along the entire length of the element, except for the lateral (orbital) margin, which is smooth. This ornamentation is interrupted by a larger groove situated in the posterior third of the element as preserved, and running from anterolateral to posteromedial. This groove separates the frontal shield from the frontoparietal shield. The medial margin of the element is straight, corresponding to the middle suture between two paired frontals. In ventral view, a moderately well‐developed frontal cranial crest is visible, but it is unclear if its anterior margin corresponds to its anteriormost end or if the element is broken at that level. Posteriorly, a triangular notch corresponds to the facet for the frontal tab of the parietal. A second bone fragment presents the same ornamentation, but it is, as preserved, much shorter. Because the posterior end shows a broken end, it is not possible to stablish if the actual length was comparable to that of the frontal or not. In any case, the preserved portion shows a well‐developed ornamented shield that, again leaves a lateral smooth margin. The medial margin of the bone is straight, again suggesting a paired element, and with the medial margin of the shield following the same course. On the opposite side, the ornamented shield runs from posterolateral to anteromedial, becoming gradually narrower anteriorly. The tooth‐bearing bone with one broken tooth (IPS49841) is poorly preserved, but it shows that dentition was well‐spaced, subpleurodont, with a wide base and a much thinner crown (not preserved). A basal foramen is situated posteroventrally. The subdental (if the element is correctly interpreted as a dentary) shelf is thin, poorly developed, and does not bear a sulcus dentalis. The alveolar surface is inclined, not forming a steep angle between the subdental shelf and the dentary wall. Osteoderms are poorly‐preserved and their morphology is thus difficult to assess. They present, however, the typical vermiculate ornamentation of anguines, and at least some of them are externally keeled. A series of lizard elongate vertebrae are assigned to indeterminate anguines too (description based on IPS128807, Figure 2e): caudal vertebra measuring 3.5 mm and showing an autotomal septum dividing the broken pleuraphophysis. The prezygaphophysis is elyptical, oriented from anterolateral to posteromedial, and its medial margin is in line with the lateral margin of the centrum in its mid‐length. The condyle and cotyle are rounded, but clearly dorsoventrally depressed, with a more flattened ventral margin. The posterior portion of the centrum is missing, so the shape of the neural spine and poszygapophysis is unknown. It is evident, however, that neural spine extent did not reach anteriorly the longitudinally mid portion of the centrum. Ventrally, the pleurapophysis is situated more anteriorly than the articulation of the (missing) postzygaphophysis, leaving a gap between both structures. The ventral surface of the centrum has parallel margins (after the pleurapophysis) and shows two lateral ridges and a marked central depression that runs longitudinally. The haemapophyses were probably fused, although they are broken at their bases. Laterally, the centrum looks arched, with a concave profile, but this is most probably the result of deformation and/or crushing. The latter also precludes a determination of the ratio between the height of the neural arch and that of the cotyle.

### 4.8 Remarks

The particular sculpture of both cranial elements and of the partial body osteoderms shows the presence of anguine lizards in the assemblage, although it is not possible to state if they all belong to a single taxon. The frontal from Mazaterón resembles that of the extant *Ophisaurus* and *Anguis* in the presence of a lateral smooth margin, which is absent in *Pseudopus* (Klembara, 2015; Klembara et al., 2017), a taxon whose record starts in the earliest Miocene. The condition in the Paleogene *Ohisauromimus* Čerňanský et al. 2016, *Helvetisaurus* Augé, 2005 and *Headonillia* Klembara & Green, 2010, is unknown due to the lack of frontal material, but the parietal of the latter shares the same type of ornamentation. Given the scarcity of material and its fragmentary status, we refer the material herein described to an indeterminate anguine. The clearly depressed vertebrae lacking a precondylar constriction, and showing an autotomal septum, and possibly fused haemapophysis, again hints at Anguinae (rather than Glyptosaurinae, that have nondepressed vertebrae). Among anguines, several of the features described above (elongate caudal vertebra with caudal autotomal septum, a depressed cotyle and condyle, medial margin of the prezygapophysis only reaching the lateral margin of the centrum and nonoverlap between pleurapophysis and postzygaphysis), plus its small size, again discard the possibility that these vertebrae belonged to *Pseudopus* (e.g., Čerňanský et al., 2019). This material compares better with *Ophisaurus* caudal vertebrae, but the presence of other anguines in the Paleogene for which the morphology of caudal vertebrae is poorly known makes referral beyond Anguinae impossible.

Glyptosaurinae Marsh, 1872

Glytposaurini Sullivan, 1979

Glyptosaurini indet.

Material: IPS49833‐49835, IPS128795‐128797, IPS128805‐128806, osteoderms (Figure 2g–i).

Description: The most abundant elements of glyptosaurines in the assemblage are osteoderms (IPS49833‐49835, IPS128795‐128797, IPS128805‐128806, Figure 2g–i). At least on one occasion the osteoderm is fused to the external surface of an indeterminate skull bone (Figure 2g). With the exception of IPS128809 described below, all correspond to thick, polygonal osteoderms with large tubercles on their external surface, and strongly interdigitated suture margins. On some occasions, the presence of turbercles is not so evident (e.g., IPS128797), but this observation usually corresponds to osteoderms with a heavily weathered surface, most probably due to digestion processes.

### 4.9 Remarks

The description above fits the characteristic shape of osteoderms of members of the tribe Glyptosaurini, mainly regarding the presence of polygonal osteoderms bearing tubercles on their external surface (e.g., Buffrénil et al., 2010; Estes, 1983; Sullivan, 1979, 2019). It is not possible, however, to assign material to a particular genus with the material at hand.

“Melanosaurini” Sullivan, 1979

cf. “Melanosaurini” indet.

Material: IPS128809, osteoderm (Figure 2j).

Description: A single glyptosaur ostoderm (IPS128809, Figure 2j) stands out among the rest in being much thinner, flatter, and with smaller tubercles. It is only partially preserved, but some of its margins are straight and suggest an extensive lateral contact, like in skull osteoderms.

### 4.10 Remarks

If properly interpreted as a skull osteoderm, it seems to belong to a different glyptosaurine than the material referred above to Glyptosaurini indet., because it differs in the lack of a clearly hexagonal‐polygonal shape, in being much thinner and smaller, in lacking the strong interdigitations seen in lateral margins of Glyptosaurini skull osteoderms, and in bearing much smaller tubercles. This osteoderm compares much better with those seen in contemporaneous members of the likely paraphyletic assemblage known as “Melanosaurini,” and more specifically, to the osteoderms present on the maxilla of for example, *Paraplacosauriops*, from the middle or late Eocene of France (Augé & Sullivan, 2006; Georgalis, Čerňanský, & Klembara, 2021). In any case, even if referral to “Melanosaurini” (i.e., a non‐glyptosaurini glyptosaurine) is tentative, this osteoderm morphology points to the presence of a second glyptosaurine at Mazaterón.

Anguidae indet.

Material: IPS128803, partial premaxilla (Figure 2b); IPS128810, anterior portion of right dentary.

Description: IPS128803 (Figure 2b) is a partial premaxilla preserving only one tooth. The bases for three additional teeth are preserved, but the tooth count was likely higher, according to the portion of the maxillary processes that is missing. The preserved tooth is conical and bears a unicuspid and pointed crown. It also shows a slight inward curve. Although partially masked by a displacement of the tooth, it is clear that there was no central enlarged tooth. Above the base of these teeth the premaxilla bears two processes (the right one is broken), and a large foramen in the middle. The nasal process is wide at its base, but it is broken, so its dorsal extension cannot be determined. IPS128810 is an anterior portion of right dentary without preserved teeth. The four observable tooth bases (attachment portion) show that the teeth reached the end of the poorly developed subdental shelf, which did not form an angle with the dentary wall, but rather an inclined and continuous surface. Ventrally, a rather deep groove probably represents the anterior end of the open Meckelian fossa. There is not an angle at the symphysis. Labially, there is a large elliptical foramen, and the anterior portion of a second one.

### 4.11 Remarks

Both elements present the typical anguimorph subpleurodont implantation. Among anguimorphs, the lack of plicidentine and tooth morphology are against platynotan affinities. Concerning the rest of anguimorphs, anguids are by far the most abundant anguimorphs in contemporaneous European localities, and the only ones known from other material at Mazaterón.

Indeterminate lizards.

Material: IPS128812, fragment of tooth‐bearing bone; IPS128815, indeterminate fragment of tooth‐bearing bone; IPS128811, isolated unicuspid tooth.

IPS128812 is a fragment of tooth‐bearing bone (most likely a maxilla) preserving a single tooth and the bases of two additional teeth. The preserved tooth is rather uninformative, being unicuspid and with a subtle striation. IPS128815 is an indeterminate fragment of bone bearing one complete and one broken tooth. The morphology these two specimens is compatible with that of the generalized scincid described above (morphotype 2), but they are too poorly preserved to be formally referred to the same taxon. IPS128811 is an isolated unicuspid tooth, with a presumably pleurodont implantation. It is poorly informative beyond the fact that it has long culmen lateralis.

### 4.12 Remarks

In all cases, these specimens are poorly diagnostic, but they could represent a scincid (first two specimens) and the anterior dentition of a lacertid (third one). However, due to poor preservation, they are best regarded as indeterminate lizards.

## 5 DISCUSSION

The locality of Mazaterón, regarded as corresponding to the MP16 level (Badiola et al., 2022), is not just one of the most important vertebrate localities of the entire Iberian Paleogene, but it is also one of the few middle Eocene sites for which a diverse assemblage of lizards and amphisbaenians is available. Studies on the squamates from other roughly contemporaneous localities from Spain stored at the ICP (e.g., Caenes in Salamanca, and Sant Jaume de Frontanyà, in Catalonia) are under progress, and the assemblage from the locality of Pontils (Catalonia) has been preliminarily studied in Minwer‐Barakat et al. (2023). The identifications provided here for the material from Mazaterón fill the gap between known early and late Eocene Iberian faunas, providing insights on the changes in the composition of lizard and amphisbaenian assemblages through time along the Iberian Eocene. They also fill a geographical gap, because Mazaterón is situated between the localities bearing previously described assemblages and situated in opposite extremes of the Iberian Peninsula (Portugal and Catalonia).

Eocene Iberian squamates almost exclusively appear in the form of disarticulated and disassociated material (e.g., Bolet, 2017; Bolet & Evans, 2013). The process of screen‐washing also influences the final state of preservation of the specimens, which usually break during the process. According to this, despite the richness in number of specimens at many localities, comparison to other Paleogene localities presenting better preserved material is not always easy. Comparison is more or less feasible with other disarticulated material (e.g., specimens coming from Phosphorites du Quercy and other screen‐washing localities, see Augé, 2005 and Georgalis, Čerňanský, and Klembara, 2021), but it is much more difficult with localities yielding articulated specimens like Messel and Geiseltal (e.g., Georgalis, 2017; Smith, 2009; Smith et al., 2018), although this is in the process of being partially addressed through the study of CT‐scanned specimens (e.g., Čerňanský & Smith, 2018, 2019). For Iberian Paleogene material, identification at the species or genus level is rarely achieved, but the recognition at a higher rank is usually possible and represents an important source of information, mainly when analyzed through time or across geography. Even at this level of uncertainty, the forms identified herein raise the number of identified members of the paleoherpetofauna from six (three turtles, two crocodiles and one ?lacertid) to 13. Besides, the squamate assemblage from Mazaterón is important in providing a milestone between the early and late Eocene described faunas so far. Regarding earlier faunas, described in Rage and Augé (2003) and Bolet (2017), it shows the persistence of pleurodont iguanians, lacertids, glyptosaurine, and anguine anguids and, possibly, scincids. On some occasions, the same genus might be regarded as possibly constantly present through the recorded Iberian Eocene (e.g., the iguanid *Geiseltaliellus* and, less certainly, the lacertid *Dormaalisaurus*).

Among the two morphotypes of cf. scincid dentition, one is rather generalized and thus poorly informative. It compares well, however, with earlier (early Eocene) Iberian material like that was described in Bolet (2017), but it is also indifferentiable from many extant scincids. The other one, however, is different from all other described Paleogene European lizards (pers. obs.), and the recovery of more complete material could lead to the recognition of a potential new taxon.

Lacertids are poorly represented at Mazaterón, and have been identified on the basis of bicuspid dentition. More specifically, material compares well with *Dormaalisaurus*, which has been tentatively recognized in the Early Eocene (Bolet, 2017) and more confidently identified in the late Eocene (Bolet & Evans, 2013) of Catalonia. If correctly identified, this would mean that the genus had a rather continuous presence in the Iberian Eocene.

Amphisbaenians are clearly recognized on the basis of dentary elements, but no vertebrae are available. The dentaries are rather generalized, and compare well with elements attributed to blanids recovered in other European Eocene localities (e.g., Augé, 2012; Bolet & Evans, 2013; Folie et al., 2013). Particularly, the amphisbaenian from Mazaterón shows some similarities (e.g., the rather widespaced tooth bases) to *Louisamphisbaena* from the MP16 of France (Augé, 2012). However, because no maxilla is preserved at Mazaterón, it is not possible to ascertain the presence of other diagnostic characters like the second recurved maxillary tooth. It is thus regarded as an inderterminate blanid. The Mazaterón record is also highly interesting in representing one of the oldest (if not the oldest) records for the group after the apparent gap (MP11–MP15) that lasted the greatest part of the middle Eocene in Europe (Augé, 2012; Rage, 2012). Mazaterón is currently assigned to the MP16, what fits well with the age of return of amphisbaenians in Europe after the mentioned gap. The lack of amphisbaenians at Pontils, if not an artifact, could support a slightly older age of this assemblage when compared to those from the MP16, including Mazaterón (see Minwer‐Barakat et al., 2023). The single Iberian late Eocene locality that has been studied in detail (Sossís, MP17a; see Bolet & Evans, 2013) also records amphisbaenians (?blanids), a group becoming widespread and abundant in the Iberian Neogene, more specifically the genus *Blanus* (e.g., Bolet et al., 2014), which has a presence in extant herpetofaunas in the western and eastern margins of northern Mediterranean since the Miocene until today (Bolet et al., 2014; Georgalis et al., 2018).

Interestingly, it seems that Mazaterón lacks acrodontan iguanians, gekkotans, and varanoids, all of them present in earlier (early Eocene) Iberian localities. However, given the limited number of recovered specimens of lizards, the possibility that taxa with a low overall occurrence (as it is usually the case for e.g., gekkotans in the Paleogene of the Iberian Peninsula) could eventually be recovered through more extensive screen‐washing cannot be discarded. Their absence could be, thus, a sampling artifact. In other cases, regarding, for example, the lack of acrodont iguanians, Mazaterón is possibly showing an actual change in composition. Acrodont iguanians (in the form of agamids) are known from many several early Eocene European localities (see Augé, 2005; Augé & Smith, 1997; Bolet, 2017; Čerňanský et al., 2023), but are apparently absent from middle and late Eocene localities, just to return in the Oligocene (Rage, 2012). A good portion of middle Eocene assemblages in Europe is represented by localities with a completely different paleoenvironmental and preservational setting (Messel, Geiseltal), a circumstance that could influence apparent changes in composition. However, agamids are also apparently absent from localities with comparable collections of disarticulated and disassociated material, like the MP14 locality (Lissieu; Rage & Augé, 2010) or later middle and late Eocene localities (Augé, 2005; Augé & Smith, 1997). According to this general absence from contemporaneous European localities, the lack of agamids in the Iberian middle Eocene (this work) and late Eocene (Bolet & Evans, 2013) might represent an actual retrieval of the group from the zone. Moreover, early Eocene and middle Oligocene agamid taxa are clearly different, supporting the idea of two different Paleogene dispersals of the group to Europe. The lack of Oligocene Iberian squamate assemblages precludes any discussion of the possible Paleogene return of the group in that particular region. The group is again recorded in the Iberian Pliocene (Delfino et al., 2008; Piñero et al., 2023) and Pleistocene, in what is interpreted as a retraction of a Miocene widespread distribution across Europe (Colombero et al., 2017; Georgalis et al., 2019; Venczel & Hír, 2015) of these and other thermophilic lizards toward southern climatic refugia before their final extirpation (e.g., Bailon & Blain, 2007; Blain et al., 2016; Georgalis et al., 2017).

Glyptosaurs are known from all Iberian Paleogene localities for which lizard assemblages have been described, although at least on one occasion, at Pontils (middle Eocene of Catalonia), they are extremely scarce (Minwer‐Barakat et al., 2023). The recovery of a multiple glyptosaur osteoderms at Mazaterón supports the interpretation that the scarcity of glyptosaur remains at Pontils could be related to local environmental factors, as suggested by Minwer‐Barakat et al. (2023), although glyptosaurs are apparently much more abundant in early and late Eocene assemblages (pers. obs.). In any case, it seems that glyptosaurs are a constant component of the Eocene faunas in the Iberian Peninsula, starting in the MP7 (Rage & Augé, 2003), continuing in younger early Eocene localities (Bolet, 2017), presenting several occurrences in the middle Eocene (Minwer‐Barakat et al., 2023; this study), and following into the late Eocene at Sossís (Bolet & Augé, 2014; Bolet & Evans, 2013) and later localities of the Pyrenees (pers. obs.). Although the presence of Melanosaurini has been reported for the Iberian Early Eocene (Rage & Augé, 2003), younger localities have only unambiguously shown the presence of Glytposaurini, as revealed by thick and polygonal skull osteoderms (Bolet & Evans, 2013; Minwer‐Barakat et al., 2023). The single flat and thin glyptosaur osteoderm described above (IPS IPS128809, Figure 2j) is the only tentative referral to a “melanosaurini” Iberian glyptosaur besides the early Eocene Portuguese material. In Europe, members of Glyptosaurinae appear in Europe by the MP7 as immigrants from North America (Sullivan, 2019), and they are constant components of European assemblages until the Eocene–Oligocene boundary, when they disappear from the continent. This contrasts with a longer survival of the group in North‐America and Asia, where they are recorded in the Oligocene as well (Čerňanský & Augé, 2019; Sullivan, 2019).

The distribution of anguines in Europe is less clear than that of glyptosaurs. Anguids have been recognized from the Iberian Late Cretaceous (Blain et al., 2010; Bolet, 2017), but the fragmentary nature of the specimens and the lack of osteoderms make an identification beyond that level difficult. Anguines have been interpreted as another example of a group reaching Europe in the early Eocene (Rage, 2012) and becoming widespread and abundant in Western Europe along the Eocene (Augé, 2005; Georgalis, Čerňanský, & Klembara, 2021) but the presence of indeterminate anguids in the Cretaceous opens the possibility that anguines could have been present before the Eocene. The lack of anguines in the scarce Paleocene European localities seems to favor the arrival of the group during the Eocene, at the same time as clear North‐American immigrants like iguanids, glyptosaurs, and helodermatids (Augé, 2003b; Rage, 2012). In any case, anguines are known from all Iberian Paleogene localities that have been studied, with records in the early Eocene (Bolet, 2017 and, tentatively, Rage & Augé, 2003), the middle Eocene (Minwer‐Barakat et al., 2023; this study), and late Eocene (Bolet & Evans, 2013; Bolet & Augé, 2014). In the particular case of Pontils, anguine remains seem to be more frequent (yet still scarce) than glyptosaur remains, in a trend that is different to the one observed for the late Eocene of Sossís (Bolet & Evans, 2013), where both types of osteoderms are counted by thousands.

Despite the valuable information provided by the specimens described herein regarding compositional changes of lizard assemblages through time, it seems that material is too limited to contribute to the discussion of the claimed endemic nature of faunas from the Western Iberian Bioprovince (e.g., Badiola et al., 2009; Cuesta Ruiz‐Colmenares, 1991), where Mazaterón is included. There are two main problems limiting discussion of this point regarding lizards. One is that the number of Iberian localities is still low, with little temporal overlap. This situation precludes direct comparisons where changes in the composition of the assemblage can be unequivocally attributed to geographical isolation, instead of being possibly affected by changes through time. The second difficulty is related to the high level of uncertainty in identifications, and the fact that most of them correspond to a high rank. As a result, faunas could appear relatively monotonous (at the level we are able to identify them), but assemblages could still contain taxa that are different at the genus and/or species, something we are not able to recognize with the available material.

Regarding the contribution of the Mazaterón squamate assemblage to constraining the age of the locality, the fragmentary nature of the specimens and the consequent open nomenclature hamper a great improvement upon the age provided by the study of mammals. However, at least one change in composition is between the assemblages of Pontils and Mazaterón deserves attention. Note that the former reference level for the MP15, La Livinière II, has been claimed to be unreliable as it seems that the collections contain material from a different locality, possibly Robiac as a result of contamination during sample processing (Comte et al., 2012). The latter authors proposed the locality of Chéry‐Chartreuve as replacement of La Livinière II to be the new reference level for the MP15, whereas Bonilla‐Salomón et al. (2016) suggested that it could be placed somewhere along the section of Sant Jaume de Frontanyà (SJF, with SJF‐3C and D being slightly older than SJF‐1), in Catalonia. The age of both Pontils and Mazaterón would possibly be intermediate between SJF‐1 and those typically referred to the MP16 (including the reference level of Robiac), although the assemblage of Mazaterón seems to contain examples of slightly more modern faunas than Pontils. In this regard, the ongoing study of the assemblages from Sant Jaume de Frontanyà (Bolet, in prep.), in combination with deeper studies of the rodent faunas from the same levels, will be particularly interesting for discussing the relative ages of all these Iberian localities. Meanwhile, what is clear is that the assemblage of Mazaterón contains some taxa not recorded at Pontils, most notably the amphisbaenian. Because amphisbaenians are relatively easy to identify on the basis of both tooth‐bearing bones and vertebrae, it seems that the lack of identified material at Pontils could be indicative of true absence. Unless this absence is the result of the particular environmental setting of Pontils (or of undersampling), this situation could be hinting at a relatively older age for Pontils, previous to the return of amphisbaenians at Mazaterón. If Mazaterón is older than other MP16 localities, then it is possibly recording the earliest occurrence of an amphisbaenian after the gap. Mazaterón also lacks many of the taxa recorded at the slightly younger locality of Sossís (e.g., gekkotans, an amblyodont lacertid, cordyliforms, and *Pyrenasaurus*; Bolet & Evans, 2013; Bolet & Augé, 2014). The presence of the latter in the late Eocene of both Catalonia and France has been interpreted (Bolet & Augé, 2014) as supporting evidence for a previously suggested connection between faunas of these areas. If *Pyrenasaurus* is actually absent from Mazaterón, its absence could be interpreted as the result of isolation of these faunas from those of the rest of Europe, although it could be also explained by other factors, like temporal or environmental differences, or undersampling at Mazaterón (note that *Pyrenasaurus* is specimens are extremely scarce even in the extremely proliferous localities where it has been identified). It is thus unclear if some of the taxa lacking at Mazaterón could be eventually recovered through more extensive screen‐washing, but it is unlikely that all these differences are artefactual. Ongoing studies are expected to fill temporal and geographical gaps through the description of new Iberian assemblages, but it might be necessary to more extensively sample key localities (including Mazaterón) in order to get a more accurate view of the evolution of small amphibians and reptiles in the Eocene of the Iberian Peninsula. Meanwhile, the Iberian Peninsula is slowly becoming a privileged area for the study of these faunas in Southern Europe, in a trend mirroring the situation of the mammal fauna. Moreover, the study of Eocene lizard assemblages, even if through rather fragmentary material, is gradually filling a gap between a better known local record in the Mesozoic (notably in the Cretaceous, see, for example, Blain et al., 2010; Blanco et al., 2016; Bolet & Evans, 2010b, 2011, 2012; Cabezuelo Hernández et al., 2022; Evans & Barbadillo, 1997, 1998, 1999; Evans & Bolet, 2016; Pereda‐Suberbiola et al., 2015; Pérez‐García et al., 2016; Rage, 1999) and Miocene (e.g., Antunes & Mein, 1981; Antunes & Rage, 1974; Bolet et al., 2014; Casanovas‐Vilar et al., 2022; Crusafont & Villalta, 1952; Delfino et al., 2013; Villa et al., 2018; Villa & Delfino, 2019), providing a more complete view of the pre‐Pliocene evolutionary history of lizards and amphisbaenians in a region that stands out for its central paleobiogeographical position between continents. As a whole, the Eocene Iberian lizard and amphisbaenian faunas match well with those of the rest of Europe, revealing a high degree of homogeneity across the continent, the faunas of which are the result of a mixture between North‐American faunas (e.g., iguanids, glyptosaurs, and helodermatids) that reached the continent in the early Eocene, taxa with a local origin (e.g., lacertids) and forms with a possible African origin (e.g., cordyliforms from the late Eocene of Sossís; see Bolet & Evans, 2013). Other groups, like agamids varanids and palaeovaranids have a more uncertain biogeographical origins (Augé, 2003b; Georgalis et al., 2017; Smith & Habersetzer, 2021), an observation that could possibly apply to anguines and scincids. In any case, faunal interchanges between Europe and Africa have been claimed on the basis of, for example, pan‐trionychid turtles (Georgalis, Čerňanský, & Mayda, 2021), what raises the potential interest of Eocene Iberian faunas as dispersal routes between these two continents. Ongoing studies of different members of the Iberian Paleogene herpetofaunas (including lizards and amphisbaenians, but also snakes and amphibians) are expected to provide a more accurate view of the environment in which the important ensemble of Iberian Eocene primates lived.

## AUTHOR CONTRIBUTIONS

**Arnau Bolet:** Conceptualization; funding acquisition; investigation; methodology; project administration; resources; validation; writing – original draft; writing – review and editing.

## CONFLICT OF INTEREST STATEMENT

The author declares no conflict of interest.

## Data Availability

All the data are available within the main manuscript.
